# Differential proteomic analysis of mouse cerebrums with high-fat diet (HFD)-induced hyperlipidemia

**DOI:** 10.7717/peerj.13806

**Published:** 2022-08-03

**Authors:** Changming Chen, Meiling Wen, Caixia Wang, Zhongwen Yuan, Ya Jin

**Affiliations:** 1 Guangdong University of Technology, School of Biomedical and Pharmaceutical Sciences, Guangzhou, Guangdong, China; 2 The Third Clinical School of Guangzhou Medical University, Department of Pharmacy, Guangzhou, Guangdong, China; 3 Guangzhou Medical University, Key Laboratory for Major Obstetric Diseases of Guangdong Province, Guangzhou, Guangdong, China

**Keywords:** High-fat diet, Hyperlipidemia, Mouse, Cerebrum, Brain, Lipid, Proteomics

## Abstract

Hyperlipidemia is a chronic disease characterized by elevated blood cholesterol and triglycerides and there is accumulated evidence that the disease might affect brain functions. Here we report on a proteomic analysis of the brain proteins in hyperlipidemic mice. Hyperlipidemia was successfully induced in mice by a 20 week high-fat diet (HFD) feeding (model group). A control group with a normal diet and a treatment group with HFD-fed mice treated with a lipid-lowering drug simvastatin (SIM) were established accordingly. The proteins were extracted from the left and right cerebrum hemispheres of the mice in the three groups and subjected to shotgun proteomic analysis. A total of 4,422 proteins were detected in at least half of the samples, among which 324 proteins showed significant difference (fold change >1.5 or <0.67, *p* < 0.05) in at least one of the four types of comparisons (left cerebrum hemispheres of the model group *versus* the control group, right cerebrums of model *versus* control, left cerebrums of SIM *versus* model, right cerebrums of SIM *versus* model). Biological process analysis revealed many of these proteins were enriched in the processes correlated with lipid metabolism, neurological disorders, synaptic events and nervous system development. For the first time, it has been reported that some of the proteins have been altered in the brain under the conditions of HFD feeding, obesity or hyperlipidemia. Further, 22 brain processes-related proteins showed different expression in the two cerebrum hemispheres, suggesting changes of the brain proteins caused by hyperlipidemia might also be asymmetric. We hope this work will provide useful information to understand the effects of HFD and hyperlipidemia on brain proteins.

## Introduction

Hyperlipidemia is a chronic disease defined by elevated blood levels of cholesterol and/or triglycerides, and might arise as a result of many risk factors, including excessive intake of high-fat foods, genetic defects and metabolic disturbances (Karam, Yang & Li, 2017). Hyperlipidemia has been characterized as a common risk factor of many cardiovascular and metabolic diseases such as atherosclerosis, heart attack, stroke, obesity and type-2 diabetes (Karam, Yang & Li, 2017). According to the latest survey (2021), there were 93 million American adults that have a total cholesterol count above the recommended limit of 200 mg/dL, and the incidence of hyperlipidemia is increasing among the populations in developing countries due to the prevalence of unhealthy lifestyles (Zhang et al., 2020; NCD Risk Factor Collaboration, 2020; Hyperlipidemia: What Is It, Causes, Symptoms, Diagnosis & Treatment, 2021). At present, treatment of hyperlipidemia generally requires a combination of drug medication and lifestyle change (Kelly, 2010; Last, Ference & Falleroni, 2011).

Besides the cardiovascular and metabolic disorders, hyperlipidemia has also been reported to affect brain functions (Pappolla et al., 2003; Rahman et al., 2005; Thirumangalakudi et al., 2008; Cao et al., 2015; Ettcheto et al., 2015; Paul & Borah, 2017; Eriksson et al., 2017; Wang et al., 2018; Uppin et al., 2020; Dimache et al., 2021; Feringa & vander Kant, 2021). Cao et al. (2015) reported that hyperlipidemia exacerbated cerebral injury of middle cerebral artery occlusion/reperfusion rats *via* oxidative stress, inflammation and neuronal apoptosis. The blood brain barrier (BBB) was observed to be compromised in hyperlipidemic animals and subsequently led to increased levels of total cholesterol (TC), total triglyceride (TG) and low-density lipoprotein (LDL)-cholesterol, and decreased high-density lipoprotein (HDL)-cholesterol in the brain (Paul & Borah, 2017; Wang et al., 2018). There have been accumulating evidence that abnormal lipid metabolism may contribute to the pathogenesis of neurodegenerative diseases such as Alzheimer’s diseases (AD) and Parkinson’s diseases and the suggested mechanisms included impairment of BBB, amyloid deposition, pathological Tau accumulation, neuroinflammation and oxidative stress in the brain (Pappolla et al., 2003; Rahman et al., 2005; Thirumangalakudi et al., 2008; Ettcheto et al., 2015; Paul & Borah, 2017; Eriksson et al., 2017; Uppin et al., 2020; Dimache et al., 2021; Feringa & vander Kant, 2021). However, not all of the research in the field found this association. For example, several cross-sectional studies showed higher TG levels were not correlated with or were even negatively correlated with cognitive impairment (Van Exel et al., 2002; Zhao et al., 2019; Dimache et al., 2021).

Proteomics is the large-scale study of all proteins in a complex biological sample, and has been used to investigate the effect of high-fat diet (HFD) on the brain for pathological mechanism studies and therapeutic target discoveries (Manousopoulou et al., 2016; Tu et al., 2017; Siino et al., 2018; McLean et al., 2019). Based on the quantitative proteomics of two-dimensional liquid chromatography (LC) and mass spectrometry (MS), Manousopoulou et al. (2016) analyzed the proteomic profile of the hypothalamus from the mice exposed to a HFD or with anorexia for 8 weeks, and the comparison revealed therapeutic targets of appetite regulation. Using two-dimensional gel electrophoresis (2-DE) followed with MS analysis, McLean et al. (2019) investigated early and reversible changes of the hippocampal proteome of mice after 3 days, 1 week and 2 weeks of a HFD, and the result showed that approximately 40% of the differential proteins were associated with AD. A partial recovery of the hippocampal proteome was also observed in mice that were fed a HFD for 1 week and returned to a low-fat diet for a further week. In a work investigating the impact of the consumption of different obesogenic diets to the mouse brain phosphoproteome, the authors reported that phosphorylation of proteins involved in synaptic plasticity and brain function were changed by a 12-week HFD feeding (Siino et al., 2018). Tu et al. (2017) used 2-DE and MS to identify new biomarkers of cerebral arteriosclerosis in hyperlipidemic rabbits fed a HFD for 12 weeks, and found that eight proteins showed differential expression in intra- and extracranial cerebral arteries. These works showed proteomics could provide new information on the study of HFD or hyperlipidemia effects on brain, but possibly due to the differences in the employed animal models, analysis methods and data interpretation preference, it is difficult to correlate the information to get a relatively comprehensive view.

To investigate the effects of hyperlipidemia on brain proteomes, a mouse model of hyperlipidemia was developed with a 20-week HFD feeding in this work. The cerebrums of three animal group, *i.e.*, control (normal diet), model and treatment, were submitted to shotgun proteomic analysis. The treatment group (SIM group) was set up by giving simvastatin to the HFD-fed mice as it is one of the most commonly used drugs to reduce blood lipids (Murphy et al., 2020). Given the asymmetry of brain structure and function, the cerebrums were split into left and right hemispheres and were analyzed separately. A total of 4,422 proteins were identified and quantified, among which 95, 110, 102 and 75 proteins were detected to show statistic difference in four types of comparisons, *i.e.*, left cerebrums of the model group *versus* the control group, right cerebrums of model *versus* control, left cerebrums of SIM *versus* model and right cerebrums of SIM *versus* model, respectively (fold change >1.5 or <0.67, *p* < 0.05). These dysregulated proteins, totally 324, could be classified into several categories according to their functions and pathophysiological roles. The two major categories involving lipid-related processes and brain function-related processes were discussed in detail.

## Materials & Methods

### Materials

Tris and glycine, grade for electrophoresis, were purchased from Bio-Rad (Hercules, CA). Porcine sequencing-grade modified trypsin was from Promega (Madison, WI). Pre-stained Mw protein markers for gel electrophoresis (10–180 kDa) was purchased from Thermo Fisher Scientific (Waltham, MA). Tween-20 and Tris-buffered saline (TBS) for molecular biology use were from Beijing Dingguo Changsheng Biotech. Co., Ltd. (Beijing, China). The simvastatin tablets were purchased from the Zhejiang Jingxin Pharmaceutical Co., Ltd. (Zhejiang, China). Primary antibodies for Western blotting were anti-Arc antibody (rabbit monoclonal, Product #183183; Abcam, Cambridge, UK), anti-Egfr antibody (rabbit monoclonal, #52894; Abcam, Cambridge, UK), anti-Hmgcs2 antibody (rabbit monoclonal, #137043; Abcam, Cambridge, UK), anti-Nrp2 antibody (rabbit monoclonal, Product #3366; Cell Signaling Tech., Danvers, MA, USA) and anti-GAPDH (rabbit monoclonal, Product #2118S; Cell Signaling Tech., Danvers, MA, USA). Secondary antibody was anti-rabbit IgG-Hrp (goat polyclonal, Product #AP132P; Merck, Kenilworth, NJ). The transfer membrane for blots was PVDF membrane with 0.45 µm pore size (Product #IPVH00010; Merck, Kenilworth, NJ). Chemiluminescent detection kit was from Asbio Tech. (Guangzhou, China). All the other chemicals were from Sigma (St. Louis, MO) and of grades of electrophoresis, molecular biology, protein sequencing or MS analysis. Nano-HPLC trap column (Acclaim PepMap100 C18, 5 µm, 100 Å, 100 µm i.d. × 2 cm) and separation column (Acclaim PepMap RSLC C18, 2 µm, 100 Å, 75 µm i.d. × 50 cm) were both from Thermo Fisher Scientific.

### Animals

SPF male C57BL/6J mice of 6–8 weeks old were purchased from the Guangdong Medical Laboratory Animal Center (Guangzhou, China). The mice were housed in a temperature-controlled animal room (25 ± 2 °C) under a 12 h-light/12 h-dark cycle and allowed free access to food and water. The animal experiment was approved by the Institutional Animal Ethics Committee of Guangzhou Medical University (approval number: 2018-051). After one week of acclimatization, the mice were randomly separated into three groups (*n* = 10 for each group): control, model and SIM groups. The experiment lasted for 20 weeks. The control group was fed a normal diet, and the model and SIM groups were fed a HFD (D12492, 5.24 kcal/g, 60% energy from fat). In the last month the SIM group was given intragastrically with simvastatin at a dosage of 2 mg/kg in 0.25% carboxymethylcellulose suspension once a day, with the same amount of saline given to the control and model groups. The diets were purchased from Guangdong Medical Laboratory Animal Center and the detailed composition of the HFD was provided in Table S1-1.

Body weights of all the mice were recorded once a week. At the end of the experiment, the mice were fasted for 16 h but with free access to water, and anesthetized with sodium pentobarbital (40 mg/kg weight, i.p.). The blood was collected from the abdominal cavity and centrifuged at 4,000 rpm for 15 min to obtain serum. The liver and abdominal fats were removed after sacrifice and weighed. Three mice from each group were randomly selected for the brain proteomic analysis. The brains were separated and washed in cold PBS for three times and frozen at −80 °C until use.

### Blood lipids and enzyme activities

For the serum samples (*n* = 6 for each group), the levels of LDL, HDL, TC and TG, and the activities of alanine aminotransferase (ALT), aspartate aminotransferase (AST) and γ-glutamyltransferase (γ-GGT) were determined using the commercial assay kits according to the manufacturer’s instructions (Nanjing Jiancheng Bioengineering Institute Inc., Nanjing, China).

The livers were homogenized in PBS and centrifuged at 3,000 rpm for 15 min at 4 °C (*n* = 6 for each group). The supernatants were taken out and the activity of superoxide dismutase (SOD) and the level of malondialdehyde (MDA) were measured using commercial kits (Nanjing Jiancheng Bioengineering Institute Inc.).

### Protein extraction

Mice in each group (*n* = 3, totally 9 animals) were performed proteomic analysis. Extraction of the brain proteins was performed by the following procedures. The frozen brains were thawed on ice, the cerebellums were removed and the cerebrums were incised longitudinally into left and right hemispheres on a rodent brain matrix (JieKai Seiko Electronic Co., Ltd., Dongguan, China). Totally 18 hemispheres were homogenized in a lysis buffer (2% w/v SDS, 0.1 M DTT in 0.1 M Tris-HCl, pH 7.6, 1 mL/100 mg tissue) using a bead-grinder at a 50-Hz shaking frequency for 100 s (SCIENTZ-48, Xinzhi, Inc., Ningbo, China). The homogenates were heated for 5 min at 95 °C, sonicated using an ultrasonic processor (VCX-150, Sonics & Materials, Inc., Newtown, CT) for 180 s (10-sec pulse at 30% amplitude with 20-sec interval, 18 cycles, sample tubes soaked in ice water) and then centrifuged at 20,238× g for 10 min. The supernatants were collected and stored in small aliquots at −80 °C. The protein concentration was determined using a UV–VIS spectrophotometer (Lambda 365, Perkin Elmer, Waltham, MA). Briefly, the supernatants were 100-fold diluted with water, the absorbance of the solutions at 230, 280, and 320 nm were measured and the protein concentrations were calculated with preset calibration parameters.

### Protein digestion

The protein samples were processed for trypsin digestion using the filter aided sample preparation method (Wisniewski, 2019). Briefly, each sample was diluted with 8 M urea in 100 mM Tris-HCl, pH 8.5 (UA solution) and a volume of 230 µL (100 µg protein) was loaded on to a 30-kDa Mw cut-off centrifugal filter unit (AmiconR Ultra-0.5 mL 30K, Merck). The filter was centrifuged at 14,000× g for 15 min at 20 °C (the same for the later centrifugal steps) and the concentrate was further washed by adding 200 µL UA solution followed with centrifugation. Alkylation was performed by adding 100 µL of 50 mM iodoacetamide in UA solution onto the concentrate, shaking for 1 min at 600 rpm and keeping in dark for 20 min. The filter was centrifuged and the concentrate was washed three times by adding 100 µL UA solution followed with centrifugation. The urea content in the concentrate was replaced by adding 100 µL of 50 mM NH_4_HCO_3_ followed with centrifugation, for three times. Digestion was done by adding an aliquot of 40 µL 25 ng/µL trypsin in 50 mM NH_4_HCO_3_ to the concentrate, shaking for 1 min at 600 rpm and incubated in a wet chamber at 37 °C for about 12 h. The peptides were collected by centrifugation at 14,000× g for 30 min at 20 °C. The concentrate was washed with 40 µL of 50 mM NH_4_HCO_3_ and centrifuged at 14,000× g for 20 min at 20 °C. The filtrate was combined with the earlier portion. The peptides were acidified with TFA to pH 3∼4, divided into small aliquots and dried by vacuum centrifugation (Concentrator plus, Eppendorf, Hamburg, Germany). To determine the concentration, the peptide sample was resuspended with water and then the UV absorbance was measured and the concentration was estimated using the same method for protein samples. The dried peptide samples were stored at −80 °C until analysis.

### Nano-HPLC-MS/MS

The peptide samples were reconstituted with 1% v/v formic acid/5% v/v acetonitrile (ACN) to a final concentration of 65 ng/µL. For each sample, 2 µL of the solution (130 ng peptide) was loaded for nano-HPLC separation on an Ultimate 3000 RSLCnano system (Thermo Fisher Scientific). Mobile phase (MP) A was 0.1% formic acid in water and MP B was 0.1% formic acid in 100% ACN. The peptides were firstly loaded on to the trap column with MP A at 15 µL/min and allowed to bind for 5 min. Meantime the analytical column was equilibrated with 2% B at 0.4 µL/min. Then the peptides were eluted from the trap column and separated in the analytical column at 0.4 µL/min with a 120-min mobile phase-gradient as follows; ramp from 2% to 17% B over 60 min, ramp from 17% to 25% B over 30 min, ramp from 25% to 37% B over 10 min, then ramp to 80% over 5 min and hold for 5 min, then ramp back to 2% B over 1 min and hold for 9 min. The column and sampler chambers were maintained at 60 °C and 8 °C, respectively. Online MS measurement was performed with a Q-TOF mass spectrometer coupled with trapped ion mobility spectrometry (timsTOF Pro, Bruker Daltonik, Bremen, Germany). MS signals were acquired in positive ion mode over an m/z range of 100–1,700. Data-dependent acquisition (DDA)-parallel accumulation-serial fragmentation (PASEF) mode (Silveira et al., 2017) was used following the manufacturer’s recommendations with optimized parameters as follows; TIMS ramp time of 100 ms from 0.8 to 1.4 V.s/cm^2^, duty cycle locked to 100%, 7 PASEF scans for each TIMS-MS, precursor ion MS repetition as 1 and cycle overlap as 3. Target intensities of 9,000 and 2,500 were set for precursor and fragment ion accumulation, respectively. Three replicate LC-MS/MS runs were performed for each sample, resulting in a total of (3 animal groups × 3 animals × 2 hemispheres × 3 LC-MS/MS technique replicates =) 54 LC-MS/MS runs.

### Data processing and bioinformatics

The LC-MS/MS data were analyzed using MaxQuant (ver. 1.6.10.43) and the main parameters were set as follows; search type as TIMS-DDA, fixed modification as carbamidomethylation at Cys, variable modifications as oxidation at Met and N-terminal acetylation, first and main search mass tolerance both as 20 ppm, digestion specificity as trypsin/P and allowing 2 missed cleavages, database using Swiss-Prot for *Mus musculus* (2020 Jan., 17,027 sequences), “match between runs” being enabled and LFQ being used for label-free quantitation (Cox et al., 2014; Tyanova, Temu & Cox, 2016). Totally 5,479 protein groups were identified and quantified (Table S2-1). All the raw data and the MaxQuant search files have been deposited in ProteomeXchange *via* iProX partner repository (https://www.iprox.cn/) (Ma et al., 2019) with the identifier PXD030691 and the subject ID IPX0003916000.

The MaxQuant results were then processed by removing the entries that matched with reverse sequences or only identified by site. Further, only proteins detected in at least 50% of the total runs, totally 4,422 protein groups (Table S2-2), were subjected to statistical analysis. Four types of comparisons were performed on the quantities of the proteins, including left cerebrums of model *versus* control, right cerebrums of model *versus* control, left cerebrums of SIM *versus* model, right cerebrums of SIM *versus* model, respectively. Fold-change ratios and student’s *t*-test *p* values were calculated to reveal the dysregulated proteins (fold change > 1.5 or < 0.67, *p* < 0.05). A total of 324 proteins showed dysregulation in at least one of the four comparisons. DAVID (https://david.ncifcrf.gov, ver. 6.8) and PANTHER (http://pantherdb.org/, ver. 16.0) were used for functional annotations.

### Western blotting

Western blotting of the brain proteins was performed as previously reported (Wen et al., 2019). Briefly, the protein samples were thawed and supplemented to have final concentrations of 4 or 6.15 µg/µL protein, 2% w/v SDS, 100 mM DTT and 12% w/v sucrose in 0.01 M Tris-0.02 M glycine buffer (pH 9.0). Slab SDS-PAGE gels (38 mm wide × 43 mm high × 1 mm thick, 4.2–17.85% T linear gradient and 5% C, containing 1% w/v SDS) were used. Protein samples (40 or 80 µg protein/lane) and pre-stained Mw markers (4 µL/lane) were separated at constant current mode at 10 mA/gel in 0.1% w/v SDS-0.05 M Tris-0.10 M glycine (pH 9.0) until the 10-kDa marker bands reached to the gel bottom (∼60 min). The proteins were transferred onto PVDF membranes at 260 mA constant current for 120 min at 4 °C. The membranes were blocked at room temperature with 5% w/v bovine serum albumin (BSA) in a TBS-buffer (150 mM NaCl in 50 mM Tris–HCl, pH 7.4) for 1.5 or 2 h with gentle shaking. Probing with the primary antibodies (anti-Arc 1:1000, anti-Egfr 1:4000, anti-Hmgcs2 1:500 and anti-Nrp2 1:1000, all diluted in 2.5% w/v BSA TBS containing 0.1% v/v Tween-20) was performed at room temperature for 1 h or overnight at 4 °C with gentle shaking. The membranes were washed with TBS-Tween for four times (8 min each) and then incubated with the secondary antibody (anti-rabbit IgG-Hrp 1:10000, in 2.5% w/v BSA in TBS-Tween) at room temperature for 0.5 h. After washing again with TBS-Tween for four times, the blots were developed using a chemiluminesence kit and imaged with a ChemiDoc Touch Imaging System (Bio-Rad). For the detection of the reference protein glyceraldehyde-3-phosphate dehydrogenase (GAPDH), the membranes were stripped using 0.4 M NaOH and re-probed with anti-GAPDH antibody (1:5000 in 2.5% w/v BSA in TBS-Tween). The blot images were analyzed using Image-Lab (ver. 3.0) and ImageJ (ver. 1.52a).

### Statistical analysis

Statistical analysis of both the biochemical assay and Western blotting results were performed using the software GraphPad Prism version 8.0 (GraphPad Software, San Diego, CA, USA). Comparison between two groups was performed using independent-samples t test (two-tailed). Differences with *p* < 0.05 were considered statistically significant.

For the proteomic analysis results, fold changes were calculated with the mean quantities of the proteins in the two groups for comparison (four comparisons: left cerebrums of model *versus* control, right cerebrums of model *versus* control, left cerebrums of SIM *versus* model and right cerebrums of SIM *versus* model). An *F*-test was firstly performed to test if the variances from the data of the two groups were equal. Then a two-tailed, unpaired student’s test, modified for the proteins that showed unequal variances, was performed to examine if there was statistical difference between the data. Proteins with fold changes >1.5 or <0.67 and *p* < 0.05 were considered to be up-regulated and down-regulated, respectively.

## Results

### Body weight and organ index

**Figure 1:**
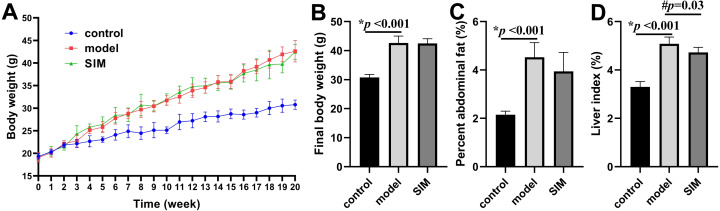
Body weight and organ index. (A) Changes of body weight over time. (B) Final body weight. (C) Percent abdominal fat, calculated by the formula [(abdominal fat weight/ body weight) ×100%]. (D) Liver index, calculated by [(liver weight/body weight) ×100%]. All the values were mean ± standard deviation (SD) (*n* = 6 in each group). *p < 0.05 comparing the model group with the control group; #*p* < 0.05, comparing the simvastatin-treated (SIM) group with the model group. The exact *p* values were given in the figure.

During the 20-week experiment, the mice in the control group were fed a normal diet and those in the model and SIM groups a HFD. The mice in the SIM group were treated with simvastatin during the last month (2 mg/kg/day). The body weight was recorded each week and the changes were presented in Fig. 1A. All the three groups showed body weight increase with prolonged feeding time, but the increase of the model and SIM groups was obviously faster than that of the control group. The curves of the model and SIM groups largely overlapped, indicating the last-month treatment did not slow down the body weight increase. Figure 1B confirmed the final weight (at the 20th week) of the mice in the model group was significantly higher than that in the control group, but no statistical difference was observed between the model and SIM groups. A similar tendency was shown for the percent abdominal fat (Fig. 1C). The liver index was also compared; the one of the model group was significantly higher than the control group, while the one of the SIM group was lower than the model group, suggesting a possible treatment effect of simvastatin (Fig. 1D).

### Blood lipids

**Figure 2:**
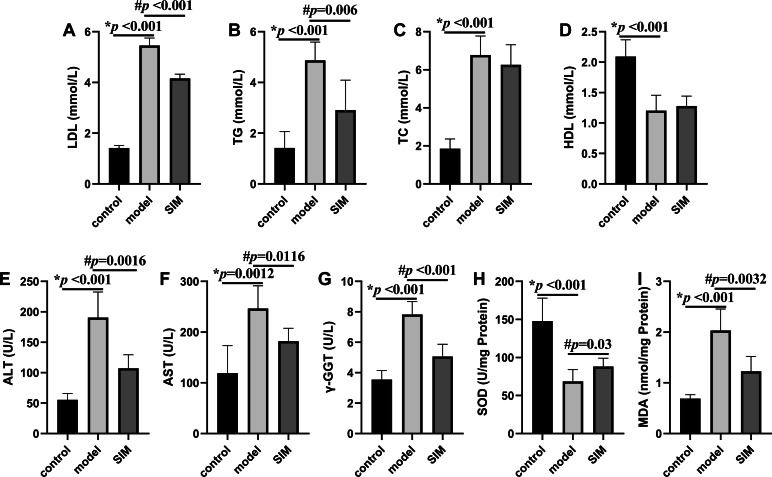
Biochemical analysis. (A) Serum low-density lipoprotein (LDL). (B) Serum triglyceride (TG). (C) Serum total cholesterol (TC). (D) Serum high-density lipoprotein (HDL). (E) Serum alanine aminotransferase (ALT). (F) Serum aspartate aminotransferase (AST). (G) Serum γ-glutamyltransferase (γ-GGT). (H) Liver superoxide dismutase (SOD). (I) Liver malondialdehyde (MDA). All the values were mean ± SD (*n* = 6 in each group). *p < 0.05 comparing the model group with the control group; #*p* < 0.05, comparing the SIM group with the model group. The exact *p* values were given in the figure.

Hyperlipidemia is characterized by the increased levels of TC, TG and LDL, and decreased levels of HDL in serum (Karam, Yang & Li, 2017). These were all observed on the mice of the model group in this work, with the levels of LDL, TG and TC drastically elevated by 2.9, 2.4 and 2.7 times, respectively, and that of HDL decreased by 0.42 times, when compared with the control group (Figs. 2A–2D). For the SIM group, the levels of both LDL and TG were lowered when compared with the model group, for 0.25 times and 0.40 times, respectively, while the levels of TC and HDL were not statistically altered. Statins are the mostly used drugs for hyperlipidemia treatment, by competitive inhibition of 3-hydroxy-3-methylglutaryl-coenzyme A reductase, an enzyme involved in the rate-limiting step of cholesterol biosynthesis (Murphy et al., 2020). This result suggested the medication in the last month partially reversed the HFD-induced increase of the blood lipids.

### Measurement of liver enzymes and oxidative stress parameters

Liver is essential for the maintenance of systemic lipid homeostasis. In this work, several markers of liver functions were measured, including serum ALT, AST and γ-GGT activities, and liver SOD activity and MDA level. As shown in Figs. 2E–2I, the activities of serum ALT, AST, γ-GGT and liver MDA were significantly increased in the model group when compared with the control group, and meantime the liver SOD activity was significantly decreased, all indicating the induction of liver damage on feeding with HFD. These changes were reversed when the HFD-fed mice were treated with simvastatin.

These results, as well as the elevated body weight and organ index, and the changes of the blood lipid levels, generally matched with the results reported for HFD-induced hyperlipidemic mice (Park et al., 2006; Gallou-Kabani et al., 2007; Lee et al., 2015; Yang et al., 2016; Zhong et al., 2018; Lee et al., 2019), showing the model was successfully developed. All the data for Figs. 1 and 2 were provided in Table S1.

### Proteomic analysis

**Figure 3:**
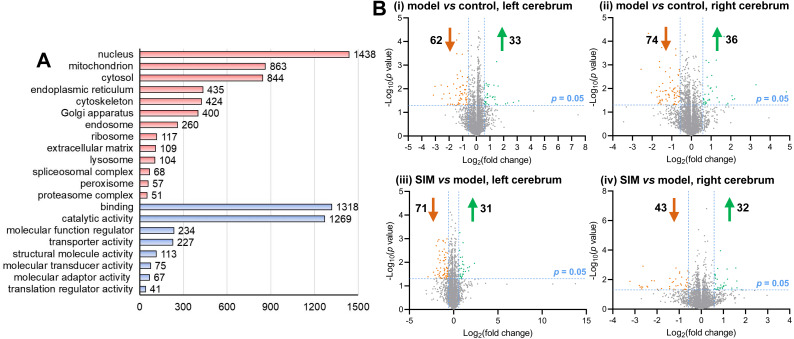
Categorization and differential analysis of the 4,422 proteins. (A) Categorization by cellular components (light red) and molecular functions (light blue), using DAVID and PANTHER, respectively. The number of the proteins in each category was given at the right. (B) Volcano plots showing the log_2_ (fold change) *versus* −log_10_ (*p* value) of each protein, for (i) the left cerebrums of model group *versus* control group, (ii) the right cerebrums of model group *versus* control group, (iii) the left cerebrums of SIM group *versus* model group, and (iv) the right cerebrums of SIM group *versus* model group. The proteins with fold change >1.5 and *p* < 0.05 were indicated in green dots (up-regulated) and those with fold change <0.67 and *p* < 0.05 were indicated in orange dots (down-regulated).

A proteomic analysis was then performed to examine the possible changes of the cerebrum proteins caused by hyperlipidemia. The cerebrum hemispheres of the mice from the three groups, *i.e.*, control, model and SIM (*n* = 3 per group), totally nine left hemispheres and nine right hemispheres, were subjected to the shotgun proteomic analysis, each with three LC-MS/MS technical replicates. A total of 4,422 proteins were detected in at least 50% of the 54 LC-MS/MS experiments (Table S2-2). Scatter plots of the detected quantities of the proteins on log_2_ scale were drawn between the LC-MS/MS experiments and good correlation was observed for all the pairs of the comparisons (Pearson coefficient 0.936–0.968) (Table S3, with coefficients between LC-MS/MS technique replicates and biological replicates marked in different colors). These 4,422 proteins were then submitted to gene ontology analysis, as shown in Fig. 3A and Table S4. The major categories assigned by cellular component were nucleus (1,438 proteins), mitochondrion (863), cytosol (844), endoplasmic reticulum (435), cytoskeleton (424) and Golgi apparatus (400). As for molecular functions, the most abundant classes were catalytic activity and binding proteins, and then regulatory and transporter activity.

**Figure 4:**
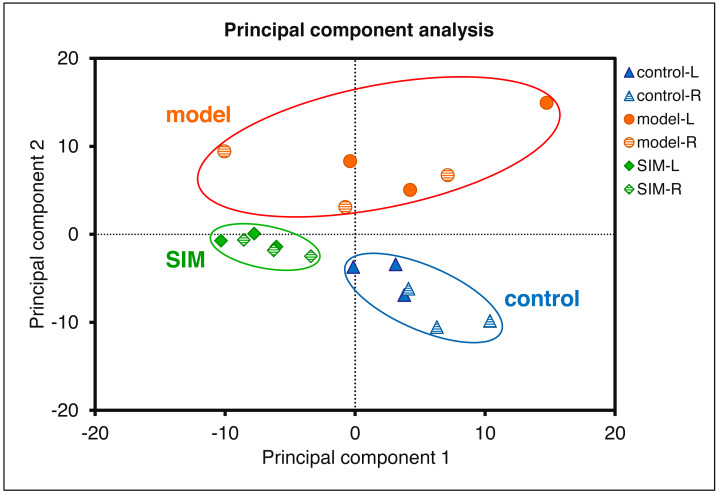
Principal component analysis of the 324 dysregulated proteins. The mean values of the technical replicates (*n* = 3) were used for each biological sample. The “L” represented for the left cerebrums, “R” for the right cerebrums.

Statistical analysis was then performed to reveal the dysregulated proteins. Four types of comparisons were performed, between the left cerebrums of the model and the control groups, the right cerebrums of the model and control groups, the left cerebrums of the SIM and model groups, and the right cerebrums of the SIM and model groups, respectively. As shown in Fig. 3B, with the criteria of fold change >1.5 or <0.67 and *p* < 0.05, among the 4,422 proteins, 62 proteins were found to be down-regulated and 33 proteins were up-regulated when the left cerebrums of the model group were compared with the control group (i). For the right cerebrums in the model group *versus* the control group, 74 proteins were down-regulated and 36 up-regulated (ii). For the left cerebrums in the SIM group *versus* the model group, the numbers of the down-regulated and up-regulated proteins were 71 and 31, respectively (iii). For the right cerebrums in the SIM group *versus* the model group, the numbers of the down-regulated and up-regulated proteins were 43 and 32, respectively (iv). Altogether 324 proteins showed dysregulation in at least one of the four comparisons (analysis procedures and results provided in Table S5-1). The principal component analysis result of these proteins showed clear separation between the samples of the control, model and SIM groups, though the data from the different hemispheres within each of the three animal groups somewhat overlapped (Fig. 4).

### Validation of proteomic results by Western blotting

**Figure 5:**
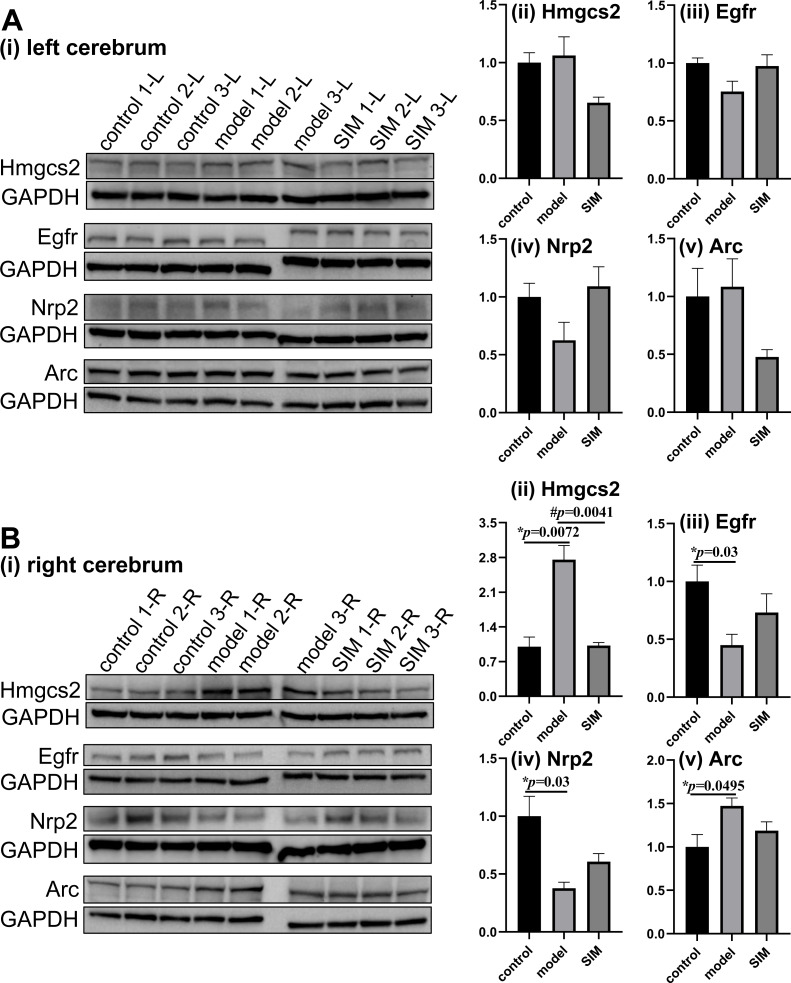
Validation of proteomic results by Western blotting. The levels of hydroxymethylglutaryl-CoA synthase, mitochondrial (Hmgcs2), epidermal growth factor receptor (Egfr), neuropilin-2 (Nrp2) and activity-regulated cytoskeleton-associated protein (Arc) were examined in (A) the left and (B) the right cerebrum hemispheres of the mice (*n* = 3 in each group). The blots are given in (i) and the densitometric values are given in (ii) to (v), expressed as mean ± standard error of the mean normalized to GAPDH (*n* = 3 in each group). *p < 0.05 comparing the model group with the control group; #*p* < 0.05, comparing the SIM group with the model group. The exact *p* values were given in the figure.

Western blotting was performed to validate the differential analysis results. Four proteins, two involved in lipid metabolism and two in brain functions and with MS-detected abundance in different ranges among the 4,422 proteins (Table S2), were examined as shown in Fig. 5. The shotgun analysis results of the four proteins were provided in Table S5 and Table 1. The original images of the blots (entire membranes) of all the samples were provided in Fig. S1.

**Table 1:** The list of the lipid processes-related and brain processes-related proteins dysregulated in the cerebrums of the hyperlipidemic mice. The table listed the lipid processes-related and brain processes-related proteins that showed dysregulation in at least one of the four comparisons, *i.e.*, between the left cerebrums of the model and control groups (M-L/C-L), the right cerebrums of the model and control groups (M-R/C-R), the left cerebrums of the SIM and model groups (SIM-L/M-L), and the right cerebrums of the SIM and model groups (SIM-R/M-R). The fold changes of the four comparisons were given in the last four columns. The “ ↑” represented up-regulation and “ ↓” represented down-regulation. The fold change >1.5 and *p* < 0.05 was marked with an asterisk (*) and the cell showed with bold text. The details of these statistical analysis results (*e.g.*, the exact *p* value) were provided in Table S5.

**Entry name**	**Protein name (Gene name)**	**Fold change**			
		**M-L/C-L**	**M-R/C-R**	**SIM-L/** **M-L**	**SIM-R/** **M-R**
**Lipid processes** **-related**					
EST1C_MOUSE	Carboxylesterase 1C (Ces1c)	↓0.443*	↓0.734	↑1.358	↓0.823
APOD_MOUSE	Apolipoprotein D (Apod)	↓0.836	↓0.612*	↓0.965	↓0.907
CO3_MOUSE	Complement C3 (C3)	↓0.446*	↓0.326*	↑1.373	↑1.826*
ANXA2_MOUSE	Annexin A2 (Anxa2)	↓0.762	↓0.964	↑1.510*	↑1.120
EGFR_MOUSE	Epidermal growth factor receptor (Egfr)	↓0.879	↓0.659*	↑1.105	↑1.537*
SPIN1_MOUSE	Spindlin-1 (Spin1)	↓0.593*	↓0.787	↑1.569	↑1.419
GSTM2_MOUSE	Glutathione S-transferase Mu 2 (Gstm2)	↓0.491*	↓0.852	↑1.117	↑1.339
SMYD3_MOUSE	Histone-lysine N-methyltransferase SMYD3 (Smyd3)	↓0.365*	↓0.652	↑2.196*	↑1.454
MIGA2_MOUSE	Mitoguardin 2 (Fam73b)	↓0.458*	↓0.729	↑2.009*	↑1.109
SEP15_MOUSE	Selenoprotein F (Selenof)	↓0.796	↓0.709	↑1.568	↑1.803*
SELM_MOUSE	Selenoprotein M (Selenom)	↓0.580*	↓0.915	↑1.074	↑1.346
LBR_MOUSE	Delta (14)-sterol reductase LBR (Lbr)	↓0.934	↓0.972	↓0.633*	↑1.055
MIA2_MOUSE	Melanoma inhibitory activity protein 2 (Mia2)	↓0.650*	↓0.810	↓0.568	↑1.113
KIME_MOUSE	Mevalonate kinase (Mvk)	↓0.690	↑1.094	↓0.902	↓0.611*
”-FABPH_MOUSE	Fatty acid-binding protein, heart (Fabp3)	↑1.565*	↑1.286	↑1.396	↑1.137
ATG2A_MOUSE	Autophagy-related protein 2 homolog A (Atg2a)	↑2.559	↑2.129	↓0.206*	↓0.669
SDCB1_MOUSE	Syntenin-1 (Sdcbp)	↑1.724*	↑1.447	↓0.865	↑1.140
HMCS2_MOUSE	Hydroxymethylglutaryl-CoA synthase, mitochondrial (Hmgcs2)	↑2.054	↑28.39*	↓0.115	↓0.144*
LST8_MOUSE	Target of rapamycin complex subunit LST8 (Mlst8)	↑6.588*	↑1.443	↓0.218	↓0.985
CO4A2_MOUSE	Collagen alpha-2(IV) chain (Col4a2)	↑1.135	↑1.504*	↓0.910	↓0.843
NSMA_MOUSE	Sphingomyelin phosphodiesterase 2 (Smpd2)	↑1.150	↑1.250	↓0.555*	↓0.524*
CGT_MOUSE	2-hydroxyacylsphingosine 1-beta-galactosyltransferase (Ugt8)	↑1.117	↑1.680*	↓0.899	↓0.752
DEGS1_MOUSE	Sphingolipid delta (4)-desaturase DES1 (Degs1)	↓0.802	↑1.872	↓0.322*	↓0.326
CERS6_MOUSE	Ceramide synthase 6 (Cers6)	↓0.851	↑2.137	↑1.696*	↑1.641
**Brain processes** **-related**					
MTA70_MOUSE	N6-adenosine-methyltransferase subunit METTL3 (Mettl3)	↓0.537	↓0.244*	↑3.959	↑1.688
RFOX1_MOUSE	RNA binding protein fox-1 homolog 1 (Rbfox1)	↓0.403*	↓0.828	↑1.640	↓0.543
S100B_MOUSE	Protein S100-B (S100b)	↓0.608*	↓0.657*	↑1.162	↓0.999
DAB1_MOUSE	Disabled homolog 1 (Dab1)	↓0.516	↓0.528	↑1.104	↑2.603*
SRGP1_MOUSE	SLIT-ROBO Rho GTPase-activating protein 1 (Srgap1)	↓0.315*	↓0.366	↑3.003	↑1.681
KCNQ5_MOUSE	Potassium voltage-gated channel subfamily KQT member 5 (Kcnq5)	↓0.611	↓0.563*	↓0.992	↑1.403
SBNO1_MOUSE	Protein strawberry notch homolog 1 (Sbno1)	↓0.368*	↓0.700	↑1.858	↑1.100
AGRG1_MOUSE	Adhesion G-protein coupled receptor G1 (Gpr56)	↓0.752	↓0.866	↑1.569*	↑1.005
NRP2_MOUSE	Neuropilin-2 (Nrp2)	↓0.206	↓0.291*	↑2.546	↑1.546
CIA2B_MOUSE	Cytosolic iron-sulfur assembly component 2B (Fam96b)	↓0.984	↓0.311*	↓0.884	↑1.661
SC6A5_MOUSE	Sodium- and chloride-dependent glycine transporter 2 (Slc6a5)	↓0.805	↓0.836	↓0.358*	↓0.287*
LRFN2_MOUSE	Leucine-rich repeat and fibronectin type-III domain-containing protein 2 (Lrfn2)	↓0.460*	↓0.988	↑1.211	↓0.839
PDE8A_MOUSE	High affinity cAMP-specific and IBMX-insensitive 3′, 5′-cyclic phosphodiesterase 8A (Pde8a)	↓0.932	↓0.321*	↓0.536	↑2.005*
GAN_MOUSE	Gigaxonin (Gan)	↓0.545	↓0.900	↑1.427	↓0.508*
FAT3_MOUSE	Protocadherin Fat 3 (Fat3)	↓0.474*	↓0.630	↑1.953	↑1.049
CMTR1_MOUSE	Cap-specific mRNA (nucleoside-2′-O-)-methyltransferase 1 (Cmtr1)	↓0.808	↓0.324*	↓0.766	↑3.041*
CTP5A_MOUSE	Contactin-associated protein like 5-1 (Cntnap5a)	↓0.502*	↓0.838	↑1.153	↑1.082
IQGA1_MOUSE	Ras GTPase-activating-like protein IQGAP1 (Iqgap1)	↓0.691	↓0.962	↑1.614*	↓0.933
FLRT2_MOUSE	Leucine-rich repeat transmembrane protein FLRT2 (Flrt2)	↓0.637	↓0.634*	↑1.518	↑1.675*
LZIC_MOUSE	Protein LZIC (Lzic)	↓0.811	↓0.363*	↓0.569	↑1.565
NNTM_MOUSE	NAD(P) transhydrogenase, mitochondrial (Nnt)	↓0.434*	↓0.546*	↑2.410	↑1.965
ANGT_MOUSE	Angiotensinogen (Agt)	↓0.167*	↓0.401*	↑2.121	↓0.782
SYT6_MOUSE	Synaptotagmin-6 (Syt6)	↓0.898	↓0.512*	↓0.628	↑1.128
KIRR3_MOUSE	Kin of IRRE-like protein 3 (Kirrel3)	↓0.174*	↑1.108	↑4.202	↑1.909
FMR1_MOUSE	Synaptic functional regulator FMR1 (Fmr1)	↓0.975	↑1.548*	↑1.007	↓0.954
SNP29_MOUSE	Synaptosomal-associated protein 29 (Snap29)	↓0.709	↑1.362	↓0.574	↓0.147*
SCAM5_MOUSE	Secretory carrier-associated membrane protein 5 (Scamp5)	↓0.747	↑1.527*	↓0.840	↓0.804
AKTS1_MOUSE	Proline-rich AKT1 substrate 1 (Akt1s1)	↓0.576	↑1.098	↑2.028*	↑1.043
CFA20_MOUSE	Cilia- and flagella-associated protein 20 (Cfap20)	↓0.371*	↑1.017	↑2.996*	↓0.832
LIMS1_MOUSE	LIM and senescent cell antigen-like-containing domain protein 1 (Lims1)	↓0.487*	↑1.011	↑1.457	↓0.944
P85B_MOUSE	Phosphatidylinositol 3-kinase regulatory subunit beta (Pik3r2)	↓0.487	↑1.264	↑1.844*	↓0.806
CI072_MOUSE	Guanine nucleotide exchange C9orf72 homolog (CI072)^a^	↓0.644*	↑1.118	↑1.710*	↓0.965
ARI1B_MOUSE	AT-rich interactive domain-containing protein 1B (Arid1b)	↓0.550*	↑1.386	↑1.690*	↓0.847
FABP7_MOUSE	Fatty acid-binding protein, brain (Fabp7)	↓0.873	↑1.064	↓0.313*	↓0.670
RB27B_MOUSE	Ras-related protein Rab-27B (Rab27b)	↑1.337	↓0.829	↓0.605*	↓0.998
LRFN1_MOUSE	Leucine-rich repeat and fibronectin type III domain-containing protein 1 (Lrfn1)	↑1.101	↓0.624*	↓0.918	↑1.527*
GRASP_MOUSE	General receptor for phosphoinositides 1-associated scaffold protein (Grasp)	↑2.183	↓0.366*	↓0.689	↑1.910
JPH4_MOUSE	Junctophilin-4 (Jph4)	↑1.841	↓0.996	↓0.251*	↑1.127
X3CL1_MOUSE	Fractalkine (Cx3cl1)	↑3.033*	↓0.520	↓0.556	↑1.689
NACHO_MOUSE	Novel acetylcholine receptor chaperone (Tmem35)	↑1.340	↓0.847	↓0.486*	↑1.144
EMB_MOUSE	Embigin (Emb)	↑1.146	↓0.652*	↑1.020	↑1.126
MYCB2_MOUSE	E3 ubiquitin-protein ligase MYCBP2 (Mycbp2)	↑1.507*	↓0.960	↓0.930	↑1.062
EFNB1_MOUSE	Ephrin-B1 (Efnb1)	↑2.014*	↓0.561	↓0.720	↑1.387
GFRA1_MOUSE	GDNF family receptor alpha-1 (Gfra1)	↑1.098	↓0.712	↑2.200*	↑2.057
LMTK1_MOUSE	Serine/threonine-protein kinase LMTK1 (Aatk)	↑1.067	↓0.475*	↑1.136	↑1.244
ARC_MOUSE	Activity-regulated cytoskeleton-associated protein (Arc)	↑1.092	↑1.868*	↓0.597	↓0.597*
PFD5_MOUSE	Prefoldin subunit 5 (Pfdn5)	↑1.728*	↑1.320	↓0.681	↓0.713
ACL6B_MOUSE	Actin-like protein 6B (Actl6b)	↑1.711*	↑1.067	↑1.009	↑1.221
LRRT4_MOUSE	Leucine-rich repeat transmembrane neuronal protein 4 (Lrrtm4)	↑1.632*	↑1.058	↓0.702	↓0.947
EPHA7_MOUSE	Ephrin type-A receptor 7 (Epha7)	↑1.480	↑1.352	↓0.291*	↓0.766
NOE2_MOUSE	Noelin-2 (Olfm2)	↑1.303	↑1.611*	↓0.730	↓0.850
EAA3_MOUSE	Excitatory amino acid transporter 3 (Slc1a1)	↑1.003	↑1.107	↓0.459*	↓0.934
CPLX1_MOUSE	Complexin-1 (Cplx1)	↑1.124	↑1.037	↑1.145	↑1.609*
GPC5B_MOUSE	G-protein coupled receptor family C group 5 member B (Gprc5b)	↑1.650*	↑1.135	↓0.443*	↓0.610*
NUCKS_MOUSE	Nuclear ubiquitous casein and cyclin-dependent kinase substrate 1 (Nucks1)	↑2.572*	↑1.203	↓0.443*	↓0.848
NAT8L_MOUSE	N-acetylaspartate synthetase (Nat8l)	↓0.986	↑1.795*	↓0.901	↓0.542*
LFG2_MOUSE	Protein lifeguard 2 (Faim2)	↑1.215	↑1.232	↓0.940	↓0.655*
G6PC3_MOUSE	Glucose-6-phosphatase 3 (G6pc3)	↑1.048	↑2.474*	↑1.083	↓0.490

**Notes.**

a The gene name of guanine nucleotide exchange C9orf72 homolog has not been documented in UniProtKB, so CI072 was used to represent the gene name.

Hydroxymethylglutaryl-CoA synthase, mitochondrial (Hmgcs2) is the rate-controlling enzyme of ketone body biosynthesis (Hegardt, 1999). The Western blots here showed Hmgcs2 in the right cerebrums of the model group was significantly up-regulated when compared with the control group, while the treatment of simvastatin reversed the up-regulation (Fig. 5B-ii). This matched with the proteomic results. Epidermal growth factor receptor (Egfr) is a transmembrane glycoprotein (Herbst, 2004), and the Western blots showed that Egfr was significantly down-regulated in the right cerebrums of the model group, and a partial recovery was observed after simvastatin treatment, which was generally consistent with the proteomic result (Fig. 5B-iii). Neuropilin-2 (Nrp2) and activity-regulated cytoskeleton-associated protein (Arc) were reported to be associated with brain-related processes (Gant et al., 2009; Fila et al., 2021). Both the Western blotting and proteomic results showed Nrp2 was significantly down-regulated in the right cerebrums of the model group *versus* control group, with a partial reverse after simvastatin treatment (Fig. 5B-iv). The proteomic results showed Arc was significantly up-regulated in the right cerebrums of the model group and the simvastatin treatment brought the level back, which was confirmed by the Western blots (Fig. 5B-v). In our proteomic results, the levels of the four proteins in the left cerebrums of the three groups showed a similar tendency of fold changes with that in the right cerebrums, but not of statistical significance, which agreed with the Western blotting results (Figs. 5A-ii to 5A-v). Altogether, these Western blotting results were generally in good accordance with the proteomic results.

### Biological processes of the dysregulated proteins

To understand the biological processes correlated with the proteomic changes, we examined the database (UniProtKB) records and publications for all the 324 dysregulated proteins about their functions, tissue specificity and pathological roles. These proteins could be classified into several major categories, as noted in Tables S5-2 to S5-7; 24 proteins were annotated/reported to be associated with lipid processes (lipid clearance and biosynthesis, *etc.*), 58 with brain processes (neurological disorders, synaptic events and nervous system development, *etc.*), 45 with enzyme catalysis, 47 with transport regulation, 16 with transcription regulation and 134 with others. In this article, we mainly focused on the examination and discussion of the first two categories, for two reasons, (1) the changes of the lipid processes-related proteins would reflect the status of lipid homeostasis in brain, and (2) the changes of the brain processes-related proteins would help understand the effects of hyperlipidemia on brain functions. The proteins of these two categories were summarized in Table 1.

**Figure 6:**
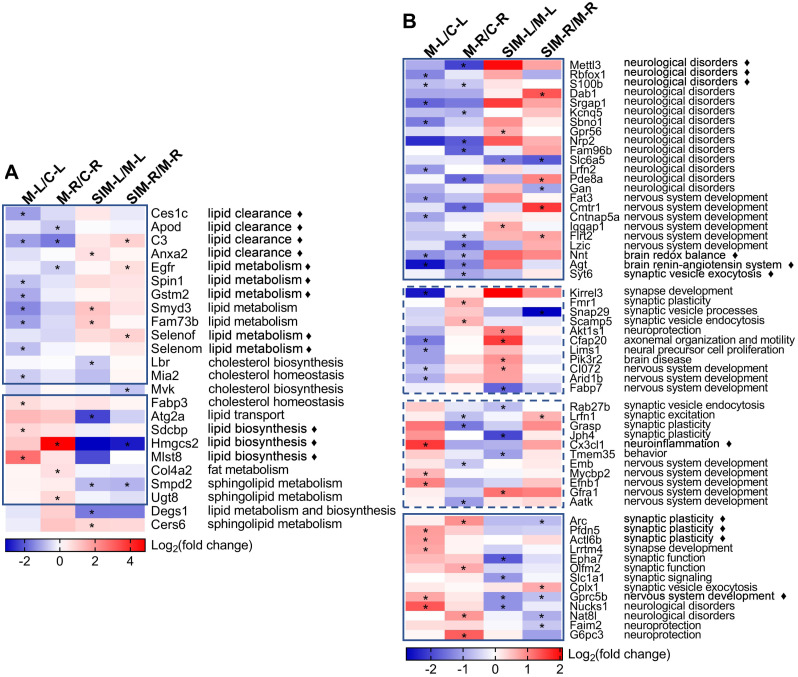
Heat maps of the lipid and brain processes-related proteins in the four types of comparisons. (A) Lipid processes-related proteins. (B) Brain processes-related proteins. Each column represents a type of comparison, and each row represents one protein (gene name used). Red indicates up-regulation and blue indicates down-regulation, and color density indicates the fold changes in log _2_ scale. The color cells marked with an asterisk (*) indicate the changes were statistically significant (fold change >1.5 or <0.67 and *p* < 0.05). The lines marked with “ ⧫” indicate these proteins were discussed in detail in Discussion. The solid-line boxes indicate the proteins that showed the same dysregulation in the two hemispheres when the model groups were compared with the control groups, *i.e.*, M-L/C-L and M-R/C-R. The dashed-line boxes indicated those that showed different dysregulation in the two hemispheres. The major biological processes based on UniProtKB records and publications are summarized on the right side for each protein. The details of these statistical results (*e.g.*, the exact fold changes and *p* values) were provided in Table S5-1.

Two heat maps were generated to show the fold changes of the 24 lipid processes-related and 58 brain processes-related proteins, respectively (Fig. 6). On the right sides the major biological processes associated with the proteins were summarized. The lipid processes-related proteins were commonly enriched in lipid clearance, cholesterol homeostasis and biosynthesis, and lipid biosynthesis and metabolism. The brain processes-related proteins were mainly enriched in neurological disorders, synaptic events and nervous system development. Detailed discussion on these proteins would be presented in the Discussion. It is interesting to note that most of the lipid processes-related proteins showed similar tendency of dysregulation in the two cerebrum hemispheres (Fig. 6A). As indicated in the solid-line boxes, the proteins in the upper one showed down-regulation (such as Ces1c, Apod, C3 and Anxa2, four proteins associated with lipid clearance), and those in the lower one showed up-regulation (such as Sdcbp, Hmgcs2 and Mlst8, three proteins associated with lipid biosynthesis), in both hemispheres in the comparisons between the model and the control groups. Also, many of them showed partial recovery after simvastatin treatment. This was also observed for about half of the brain processes-related proteins, as indicated in the solid-line boxes in Fig. 6B. But the other half of the brain processes-related proteins exhibited different dysregulation patterns in the two hemispheres, as indicated in the dashed-line boxes. For example, the levels of Kirrel3, Fmr1, Snap29, Scamp5, Akt1s1, Cfap20, Lims1, Pik3r2, CI072, Arid1b and Fabp7 decreased in the left cerebrums of the model group, while increased in the right cerebrums of the model group. When compared with the control group, the levels of Rab27b, Lrfn1, Grasp, Jph4, Cx3cl1, Tmem35, Emb, Mycbp2, Efnb1, Gfra1 and Aatk increased in the left cerebrums of the model group, while decreased levels in the right cerebrums of the model group. The changes after simvastatin treatment also showed more complex variations. It has been established that the left and right hemispheres of the mammalian brain develop with a high degree of asymmetry at both the structural and functional levels, and this asymmetry becomes more evident in the post-natal period (Cara et al., 2022). These asymmetric protein dysregulation patterns observed in this work and their corresponding biological processes suggested the changes of the brain proteins caused by the diet and lipid metabolism perturbation might also be asymmetric.

## Discussion

Many studies have reported that hyperlipidemia was a risk factor of brain dysfunction (Manousopoulou et al., 2016; Tu et al., 2017; Siino et al., 2018; McLean et al., 2019). Analysis of the brain proteome would help to gain a more comprehensive understanding about the impact of HFD or hyperlipidemia on brain protein composition and functions. In this work, a mouse model of hyperlipidemia was induced by a 20-week HFD feeding. Hyperlipidemia is generally induced in rodents with a long-term feeding from several weeks to months (Wong et al., 2016; Andreadou et al., 2020). Our results on body weight, organ index and biochemical assays (Figs. 1 and 2) showed the model was successfully established. The proteins from the two cerebrum hemispheres were then analyzed by shotgun proteomics. A total of 4,422 proteins were quantified and 324 proteins showed significant dysregulation (fold change >1.5 or <0.67, *p* < 0.05) in at least one of the four comparisons, *i.e.*, left cerebrums of model *versus* control, right cerebrums of model *versus* control, left cerebrums of SIM *versus* model and right cerebrums of SIM *versus* model. These proteins could be classified into several categories by bioinformatics analysis and two major categories involving lipid-related processes and brain function-related processes would be discussed in the following sections.

### Differences in the expression of lipid processes-related proteins

Brain is one of the most lipid-rich organs in terms of both content and diversity, and the brain lipids are essential for neuronal structures and functions. Previous studies have reported that hyperlipidemia would cause imbalance of lipid homeostasis in brain (Paul & Borah, 2017; Wang et al., 2018), and our work showed similar results (Table 1 and Fig. 6A). Twenty-four lipid processes-related proteins were found dysregulated, in at least one of the four comparisons, and the major enriched processes included clearance, biosynthesis and metabolism of lipids. As indicated with the solid-line boxes in Fig. 6A, 21 proteins showed similar dysregulation tendency in the two hemispheres when the model group was compared with the control group, *i.e.*, down-regulated (upper box) or up-regulated (lower box) in both hemispheres. The right half of the heat map also revealed that for many of these proteins, the hyperlipidemia-induced dysregulation was partially reversed with the simvastatin treatment.

In this section, we mainly discussed on 12 proteins that were reported to be involved in three kinds of lipid processes, *i.e.*, lipid clearance, biosynthesis and metabolism, respectively (indicated with diamond marks in Fig. 6A). Firstly, four proteins that participate in lipid clearance were detected to be down-regulated in the model group when compared with the control group, including carboxylesterase 1C (Ces1c), apolipoprotein D (Apod), complement C3 (C3) and annexin A2 (Anxa2). Ces1c, also known as Ces-N or Es-1, is a member of the carboxylesterase 1 family and catalyzes the hydrolysis of cholesteryl esters (Lian, Nelson & Lehner, 2018). A previous study reported that its up-regulation in the liver of type 2 diabetic rats with metformin treatment enhanced the rapid conversion of cholesterol into bile acids, thus promoted the removal of cholesterol from the body (Chen et al., 2018). In this study, its down-regulation in hyperlipidemic mice possibly suggested Ces1c mediated cholesterol clearance was impaired. Apod, a 29-kDa glycoprotein, is known as a component apolipoprotein of HDL and highly expressed in the brain. Apod belongs to the lipocalin superfamily that functions for lipid binding and transport (Dassati, Waldner & Schweigreiter, 2014). *Jiménez-Palomares et al. (2011)* reported that elevated Apod production in liver of young mice resulted in enhanced lipoprotein lipase activity and improved postprandial TG clearance, while genetic deficiency of Apod in mouse was associated with nonfasting hypertriglyceridemia and hyperinsulinemia. Acylation stimulating protein (ASP) is a fragment of C3 and was reported to stimulate TG synthesis in adipocytes. It was reported that the C3-deficient mice lacking ASP had a delayed postprandial TG, indicating the ASP pathway played an important role in postprandial TG clearance (Murray, Sniderman & Cianflone, 1999). The decreased levels of Apod and C3 suggested the hindered TG clearance might occur in brain of hyperlipidemic mice. Anxa2 was identified to be an extrahepatic inhibitor of the proprotein convertase subtilisin/kexin-9-induced LDL-receptor (LDLR) degradation. Analyses of plasma from Anxa2^−/−^ mice showed that the LDL-cholesterol levels were significantly elevated by 40% (Seidah et al., 2012). Salameh et al. (2016) reported that Anxa2^−/−^ mice had a delayed clearance of infused fatty acid (FA). They identified a FA-induced interaction between prohibitin, Anxa2 and the FA transporter CD36 and the deficiency of Anxa2 might impair the CD36-mediated transport in white adipose tissue. The decreased levels of these proteins in this work indicated that HFD-induced hyperlipidemia could disturb the lipid clearance in brain.

The expressions of the proteins related to lipid biosynthesis, such as syntenin-1 (Sdcbp), Hmgcs2 and target of rapamycin complex subunit LST8 (Mlst8), were up-regulated in the model group when compared with the control group (Table 1 and Fig. 6A). Sdcbp is a 29-kDa protein that interacts with a variety of partners, *e.g.*, membrane-bound proteins and phospholipids, through its two postsynaptic density protein 95/Discs large protein/Zonula occludens 1 (PDZ) domains. It functions *in vivo* as a multiple adaptor protein and has been implicated in various cellular events (Shimada et al., 2019). Sdcbp has also been identified to be one of the markers of extracellular vesicles (EVs) (Kowal et al., 2016), and Zhao et al. (2020b) reported that the hepatic levels of overall EVs and the markers (including Sdcbp) in mice increased in response to a HFD. Naruse et al. (2015) reported on the increased expression of Sdcbp in adipose tissue that was exposed to preeclampsia sera and they speculated it contributed to adipogenesis. In our work, the up-regulation of Sdcbp might induce increased lipid biosynthesis in brain under HFD. Hmgcs2 is known as a rate-controlling enzyme responsible for catalyzing the biosynthesis of ketone bodies (Hegardt, 1999). Ketone bodies are water-soluble lipid molecules, which can be utilized as an alternative energy source if energy provided by glucose is insufficient. Especially, ketone oxidation can be used as the main energy source in the brain tissue under a starved state (Naithani & Karn, 2020). A recent work showed that the heart level of Hmgcs2 increased in the HFD-fed mice (Li et al., 2021). Similarly, the level of Hmgcs2 increased in the liver of nonalcoholic fatty liver disease (NAFLD) mice induced by a HFD, and decreased after exercise (Qian et al., 2021). This increased level of Hmgcs2 detected in the model group agreed with the above works (Qian et al., 2021; Li, Guo & Jia, 2021), and the partial recovery after simvastatin treatment was also consistent with a report that lovastatin treatment suppressed the ketogenesis and exhibited hypolipidemic effect (Li et al., 2015). This was also confirmed by Western blotting (Fig. 5). The mechanistic target of rapamycin (mTOR) is a phosphatidylinositol 3-kinase (PI3K)-like serine/threonine protein kinase, which contains two complexes: mTOR complex 1 (mTORC1) and mTOR complex 2 (mTORC2). It was reported that mTOR has an important role in the control of lipid biosynthesis (Lamming & Sabatini, 2018). As a subunit of both mTORC1 and mTORC2 (Lamming & Sabatini, 2018), Mlst8 were detected to be up-regulated in the hyperlipidemic mice of this study. This up-regulation might promote mTOR signaling pathway-mediated lipogenesis. Also, four proteins involving in cholesterol biosynthesis or homeostasis were detected to be dysregulated in this study, including Lbr, Mia2, Mvk and Fabp3 (Table 1 and Fig. 6A, full names in Table 1). However, here their changes could not be well explained, possibly due to complex cholesterol metabolism regulation in brain.

Furthermore, our proteomic result provided a group of proteins involved in lipid metabolism that were down-regulated in the model group when compared with the control group (Table 1 and Fig. 6A). For example, Egfr, spindlin-1 (Spin1) and glutathione S-transferase Mu 2 (Gstm2) were reported to regulate abnormal lipid metabolism in various pathological processes. Egfr is a transmembrane glycoprotein with tyrosine kinase activity that can activate corresponding signaling pathways upon binding the ligands (Herbst, 2004). Two works on mouse models with NAFLD showed that inhibiting Egfr reduced fat accumulation in liver (Liang et al., 2018; Choung et al., 2019). Its roles in modulating lipid metabolism in cancers have been also reported (Bian et al., 2015; Luo et al., 2021). In this work, the down-regulation of Egfr in the right cerebrums of the model group was detected by both proteomic analysis and Western blotting. As a major maternal transcript in mice, Spin1 has been recognized as a histone methylation reader, which could epigenetically control multiple tumorigenesis associated signaling pathways (Li, Guo & Jia, 2021). Zhao et al. (2020a) reported that Spin1 modulated abnormal lipid metabolism by increasing intracellular TG, cholesterols, and lipid droplets in hepatoma cells, which enhanced the growth of liver cancer through SREBP1c-triggered fatty acid synthase signaling. In a recent work, Gstm2 was identified as a sensitive responder and effective suppressor of nonalcoholic steatohepatitis (NASH) progression, and both the mRNA and protein levels of Gstm2 were down-regulated in the liver from patients with NASH and mice fed a HFD. Mechanism study showed that Gstm2 could attenuate lipid accumulation and inflammation through the suppression of apoptosis signal-regulating kinase 1 (ASK1) and its downstream mitogen-activated protein kinase (MAPK) pathway under the metabolic stress (Lan et al., 2021). The decreased levels of Egfr, Spin1 and Gstm2 suggested the correlated lipid metabolism was harmed by hyperlipidemia in brain. This down-regulated pattern was also observed on histone-lysine N-methyltransferase SMYD3 (Smyd3) and mitoguardin 2 (Fam73b) that were crucial for lipid metabolism. Selenoprotein F (Selenof) and selenoprotein M (Selenom) are endoplasmic reticulum-resident selenoproteins that are highly expressed in brain (Pitts et al., 2013; Ren et al., 2018). Zheng et al. (2020) reported that Selenof knockout led to glucose and lipid metabolism disorders in mice. Especially in the HFD condition, Selenof knockout significantly increased TC contents in the serum and liver, and exacerbated HFD-induced obesity and hepatic steatosis. *Pitts et al. (2013)* reported that Selenom knockout mice exhibited increased white adipose tissue deposition without cognitive deficits, and this increased adiposity was likely due to diminished energy expenditure. This might match with the down-regulation of the two selenoproteins observed in this study, indicating their involvement of complex lipid metabolism in brain under hyperlipidemia.

In this study, lipid homeostasis within the brain was disturbed by the HFD-induced hyperlipidemia, as manifested by the dysregulated profiles of the lipid processes-related proteins (Table 1 and Fig. 6A). The decreased levels of the proteins functioning in lipid clearance and the increased levels of those in lipid biosynthesis detected in the model group indicated excessive lipid deposition in brain as a consequence of the HFD consumption. The down-regulation of the proteins with important functions in lipid metabolism, on another side, indicated more down-stream processes that maintain lipid homeostasis were perturbed. Moreover, as far as we know, the brain levels of Ces1c, Anxa2, Spin1, Gstm2, Smyd3, Fam73b, Selenof, Lbr, Mia2, Mvk, Fabp3, Atg2a, Sdcbp, Hmgcs2, Mlst8, Col4a2, Smpd2, Ugt8, Degs1 and Cers6 have not been reported to be correlated with HFD, obesity or hyperlipidemia (full names in Table 1). More functional analyses of other dysregulated proteins should be the direction of further research to understand the influence of hyperlipidemia on lipid events in brain.

### Changes in the expression of brain processes-related proteins

For the 58 brain processes-related proteins that showed dysregulation in this work (Table 1), their dysregulation patterns in the four comparisons and their major correlated biological processes were presented in Fig. 6B. Their changes by hyperlipidemia and the following simvastatin treatment appeared to be more diverse than the lipid processes-related proteins. We selected 11 proteins that have been frequently reported for further discussion, as indicated with the diamond marks in Fig. 6B.

Firstly, the heat map showed 23 proteins, as enclosed in the upper solid-line box in Fig. 6B, were down-regulated in both hemispheres when the model group was compared with the control group. Most of them were mainly involved in the processes of neurological disorders. For example, N6-methyladenosine (m6A) is catalyzed by the methyltransferase and plays an important role in learning and memory. N6-adenosine-methyltransferase subunit METTL3 (Mettl3) is the best known m6A methyltransferase that functions in the reversible epitranscriptome modulation of m6A modification (Zhang et al., 2018; Huang et al., 2020). It was reported that Mettl3 depletion in mouse hippocampus reduced memory consolidation ability, and hippocampal Mettl3 protein abundance had a positive correlation with learning efficacy in wild type mice (Zhang et al., 2018). Huang et al. (2020) also showed that down-regulation of Mettl3 mRNA and soluble protein levels in the human AD hippocampus may therefore correlate with the memory dysfunction associated with this disease. A similar reduction was observed on RNA binding protein fox-1 homolog 1 (Rbfox1). In a genome-wide association study on human, reduced expression of Rbfox1 was found to be correlate with increased amyloid burden and worse cognition during life, suggesting a potential role of Rbfox1 in the pathogenesis of AD (Raghavan et al., 2020). Rbfox1 is a regulator of alternative splicing events that are critical for neuronal development by binding to the (U) GCAUG element in mRNA precursors. Hamada et al. (2016) reported that knockout of Rbfox1 may induce structural and functional defects in neuronal cells, thus contributed to the pathogenesis of neurodevelopmental disorders such as autism spectrum disorder. We speculated the down-regulation of such proteins in this study might indicate an increased risk of cognitive impairment under hyperlipidemia. Protein S100-B (S100b) is a Ca^2+^-binding protein highly expressed in brain, particularly in astrocytes, and has been reported to be involved in a wide spectrum of neurological disorders such as acute brain injury and neurodegenerative diseases. The overexpression or administration of S100b often induced worsening of the diseases in both human and animal models, and neuroinflammation was found to be commonly involved in the underlying pathogenic mechanisms (Sorci et al., 2010; Michetti et al., 2019). Studies have shown that its biological activity was closely associated with its concentration, *i.e.*, S100b at low concentrations (nanomolar) exerted neurotrophic effect by stimulating the growth of neurons and enhancing their survival during development, while S100b at high concentrations (micromolar) showed toxic/proinflammatory effects (Businaro et al., 2006; Piazza et al., 2013; Niven et al., 2015; Clementi, Sampaolese & Giardina, 2016). Whether the decreased level of S100b in this study suggested its neurotrophic effect was weakened by hyperlipidemia should be further analyzed. As shown in the figure, there are more proteins that have been reported to be involved in neurological disorders were found down-regulated in the model groups in this work. This might suggest an increased risk of neurological disorders with the HFD-induced hyperlipidemia. This is also partly supported by the down-regulation of the proteins functioning in nervous system development, including Fat3, Cmtr1, Cntnap5a, Iqgap1, Flrt2 and Lzic (full names in Table 1).

Similar alterations were observed in NAD(P) transhydrogenase, mitochondrial (Nnt), angiotensinogen (Agt) and synaptotagmin-6 (Syt6). Nnt is a mitochondrial NADP-reducing enzyme responsible for the catalyzation of NADPH generation. NADPH generation catalyzed by Nnt contributes to about a quarter of the total mitochondrial NADPH pool in mouse brain (Francisco et al., 2018). Lopert & Patel (2014) reported that brain Nnt was associated with neuronal protection against oxidative stress and degeneration by coupling brain mitochondrial respiration with peroxide catabolism. Francisco et al. (2018) reported that Nnt was required for mitochondrial redox balance of brain, especially in the case of mitochondrial respiratory defects or HFD. A decreased Nnt expression in this work might reflect dysfunctional energy supply and redox balance in brain of HFD-induced hyperlipidemia. As the unique substrate of the rennin-angiotensin system (RAS), Agt could be converted into angiotensin I by renin and subsequently converted into angiotensin II by angiotensin converting enzyme (ACE) (Lu et al., 2016). It is noteworthy that brain contains an intrinsic RAS and all the components, including Agt, are produced within the brain. Brain Agt influences learning and memory mechanisms and contributes to BBB reconstitution (Cosarderelioglu et al., 2020). Mateos et al. (2011) reported that brains of mice on a cholesterol-enriched diet showed increased Agt and ACE, and they postulated that 27-hydroxycholesterol, the cholesterol metabolite which could pass BBB into brain, could be a link between high-plasma cholesterol levels and hypertension. We think this down-regulation of cerebrum Agt found in this work suggests the brain RAS could also be affected by hyperlipidemia, though it would need more work to differentiate the effect from that through the peripheral RAS. Synaptotagmins is a family of membrane-trafficking proteins that are characterized by an N-terminal transmembrane region and two C-terminal C2 domains, and there are 17 synaptotagmin isoforms in the mammals (Li et al., 1995; Dean et al., 2012). In a recent study, Wong et al. (2015) reported that neuronal activity could trigger the release of brain-derived neurotrophic factor (BDNF) by exocytosis of endosomes at postsynaptic sites. They proved this process was regulated by Syt6 with the aid of complexin and among the eight synaptotagmin isoforms that were examined, down-regulation of only Syt6 showed impaired activity-induced BDNF secretion. In this work, nine synaptotagmin isoforms were identified, but only Syt6 showed significant difference (down-regulation) in the comparison between the right cerebrums of the model and control groups (Table S2-2). This suggested hyperlipidemia might have a negative effect on the Syt6-modulated synaptic vesicle exocytosis.

After 20 weeks of the HFD, the expression of 13 proteins were up-regulated in the two hemispheres in comparison to the control group, with many showed partial recovery after simvastatin treatment, as indicated in the lower solid-line boxes in Fig. 6B. Among these proteins, many were associated with synaptic events, such as Arc, Pfdn5, Actl6b, Lrrtm4, Epha7, Olfm2, Slc1a1 and Cplx1 (full names in Table 1). For example, Arc is a master regulator of synaptic plasticity critical for learning, memory consolidation and behavior, and expressed predominantly in cortical and hippocampal glutaminergic neurons (Fila et al., 2021). The association between brain Arc levels and HFD were also reported, though the results were relatively controversial. Mateos et al. (2009) reported on the decrease of Arc in cerebral cortex and hippocampus of mice fed with a HFD and suggested the cause to be a reduced N-methyl-D-aspartate receptor (NMDAR) activity. Sable et al. (2021) also reported about the decrease of hippocampal Arc and BDNF, and poorer spatial working memory in mice fed with a high-fat, high-sucrose western diet. Ettcheto et al. (2015), on the other side, reported on the observation of cognitive impairment in hypercholesterolemic LDLR-knockout mice, but with a significant increase of Arc expression and a slight increase of NMDAR1 expression in hippocampus. Chen et al. (2020) detected the hippocampal Arc levels in HFD-fed mice and did not find significant difference from those in normal diet-fed animals. We suspect the contradiction of the results might be related to the difference of the animal models, as the former two works used 9-month HFD-fed mice, while the latter two used 5-week HFD-fed mice and 6-month hypercholesterolemia mice, respectively (Herbst, 2004; Ettcheto et al., 2015; Chen et al., 2020; Sable et al., 2021). In our study, Arc was found to be up-regulated in hyperlipidemic mice and the simvastatin treatment brought the level back to normal and the changes were significant in the right hemispheres (also confirmed by Western blotting as shown in Fig. 5). These changes suggested involvement of Arc-regulating brain processes, but whether the increase is age-related, as Ettcheto et al. (2015) suspected, would need more work. Prefoldin subunit 5 (Pfdn5) and actin-like protein 6B (Actl6b) are the other two proteins related to synaptic plasticity. Pfdn5 is predominantly localized in the pyramidal layer of CA1-CA3 regions in the hippocampus, and it was reported that the changes of its expression depended on the situation of synaptic plasticity, *i.e.*, increased in the sufficient expression of synaptic plasticity, but decreased under impaired synaptic plasticity (Kadoyama et al., 2019). Actl6b, also referred to as Baf53b, has been shown to be necessary for synaptic plasticity and long-term memory processes, possibly through the regulation of gene expression required for spine structure and function (Vogel-Ciernia et al., 2013). The lower solid-line box in Fig. 6B showed more proteins involved in synaptic events were also up-regulated by the HFD-induced hyperlipidemia in this work, supporting that synaptic structures and functions were perturbed by the disease. The up-regulated proteins also included some in other categories, *e.g.*, Gprc5b for nervous system development, Nucks1 and Nat8l involved in neurological disorders, and Faim2 and G6pc3 in neuroprotection. For example, G-protein coupled receptor family C group 5 member B (Gprc5b) is a lipid raft-associated transmembrane protein with multiple phosphorylated sites, and it was reported that Gprc5b-mediated signaling controlled the neuronal differentiation of neural progenitors and contributed to neurogenesis in the development of mouse neocortex (Kurabayashi, Nguyen & Sanada, 2013).

Furthermore, 22 brain processes-related proteins showed reverse dysregulation patterns in the two hemispheres in the comparisons between the model and the control groups, as indicated in the two dashed-line boxes in Fig. 6B, suggesting the changes of the brain proteins caused by the diet might also be asymmetric. These proteins were mainly enriched in the processes of synaptic events and nervous system development, respectively, including eight proteins associated with synaptic events (Kirrel3, Fmr1, Snap29, Scamp5, Rab27b, Lrfn1, Grasp and Jph4), and eight proteins with nervous system development (CI072, Arid1b, Fabp7, Emb, Mycbp2, Efnb1, Gfra1 and Aatk, full names in Table 1). It should be noted that the proteins in these two categories did not always have the same dysregulation patterns, *i.e.*, some showed down-regulation or up-regulation in both hemispheres (solid-line boxes in Fig. 6B) and others showed reverse dysregulation in the two hemispheres (dashed-line boxes), indicating the effects of hyperlipidemia on the processes in these two categories could be rather complex. Some other processes were also suggested to be affected by hyperlipidemia. For example, fractalkine (Cx3cl1) was detected to be dysregulated. Cx3cl1 is a well-known proinflammatory chemokine and has been reported to be altered by HFD despite of some conflicts in the published results. For example, increased level of Cx3cl1 was reported after HFD consumption and induced neuroinflammation-relevant process (Morari et al., 2014; Gonzalez-Portilla et al., 2021), while decreased Cx3cl1 expression in hippocampus and amygdala was reported in HFD-induced obese mice that showed significant cognitive deficits (Kawamura et al., 2021). Unveiling of the causes that led to the dysregulation of the protein in our study would need further studies.

The present study reported the complex changes of the brain processes-related proteins and suggested brain functions were affected by HFD-induced hyperlipidemia, especially the processes correlated with neurological disorders, synaptic events and nervous system development (Table 1 and Fig. 6B). To our knowledge, the brain levels of many proteins have not been reported to be associated with HFD, obesity or hyperlipidemia, including Mettl3, Rbfox1, Dab1, Srgap1, Kcnq5, Sbno1, Gpr56, Nrp2, Fam96b, Slc6a5, Lrfn2, Pde8a, Gan, Fat3, Cmtr1, Cntnap5a, Iqgap1, Flrt2, Lzic, Syt6, Kirrel3, Snap29, Scamp5, Akt1s1, Cfap20, Lims1, Pik3r2, CI072, Arid1b, Rab27b, Lrfn1, Grasp, Jph4, Tmem35, Emb, Mycbp2, Efnb1, Gfra1, Aatk, Actl6b, Lrrtm4, Epha7, Olfm2, Cplx1 and Gprc5b (full names in Table 1). Deeper understanding of the causes and consequences of these changes would require more information input from other disciplines such as metabolomics. It is noteworthy that 22 brain processes-related proteins were found to show different expression tendency between the two hemispheres (Kirrel3, Fmr1, Snap29, Scamp5, Akt1s1, Cfap20, Lims1, Pik3r2, CI072, Arid1b, Fabp7, Rab27b, Lrfn1, Grasp, Jph4, Cx3cl1, Tmem35, Emb, Mycbp2, Efnb1, Gfra1 and Aatk) (Table 1 and Fig. 6B, full names in Table 1), which may be explained by the brain asymmetry and diet differences.

## Conclusions

In this work, a differential proteomic analysis was performed on the cerebrum proteins with a mouse model of HFD-induced hyperlipidemia. A majority of the dysregulated proteins were involved in the processes of lipid metabolism and neurological functions. The down-regulation of the proteins involved in lipid clearance and the up-regulation of the proteins in lipid biosynthesis suggested excessive lipid accumulation in brain under hyperlipidemia. More complex dysregulation patterns were observed on the proteins reported to be involved in brain processes, but it would need further work to determine if these expression changes are correlated with brain or neurological dysfunctions. For the first time, more than 60 among these proteins were found to be altered in brain by HFD, obesity or hyperlipidemia, and further investigation on these proteins may help understanding the pathological effects and mechanisms of hyperlipidemia on brain. Moreover, twenty-two brain processes-related proteins exhibited different dysregulation patterns between the left and right cerebrum hemispheres when the model group was compared with the control group. Whether these differences suggest the effects of hyperlipidemia are brain-asymmetric would need further confirmation. Also, future work could be done by correlating the results with proteomics on the metabolic organs such as liver and pancreas. We hope this work would contribute to understand whether or how HFD and hyperlipidemia affect brain structures and functions.

## Supplemental Information

10.7717/peerj.13806/supp-1 Supplemental Information 1 Original Western blots (A) Hmgcs2. (B) Egfr. (C) Nrp2. (D) Arc. For each protein, all the (3 group × 3 animals × 2 cerebrum hemispheres =) 18 samples for the shotgun proteomic analysis were examined. The sample names were given above the lanes; “SIM” stands for simvastatin-treated group, the number that follows is the number of the animal, and the “-L” “and “-R” for left and right cerebrum hemispheres, respectively. The bands of the target proteins were indicated in the red-line frames. The molecular masses from UniProtKB (signal peptides, transit peptides and propeptides excluded; 3 kDa/N-glycosylation added) were given after the protein names.

10.7717/peerj.13806/supp-2 Supplemental Information 2 Composition of HFD and biochemical assay results

10.7717/peerj.13806/supp-3 Supplemental Information 3 Proteins detected in the cerebrum hemispheres of the HFD-induced hyperlipidemic mice

10.7717/peerj.13806/supp-4 Supplemental Information 4 Pearson correlation analysis of the proteomic quantity data

10.7717/peerj.13806/supp-5 Supplemental Information 5 The gene ontology (GO) analysis of the 4,422 proteins

10.7717/peerj.13806/supp-6 Supplemental Information 6 List of the dysregulated proteins in the cerebrums of mice with HFD-induced hyperlipidemia and the categorization by biological processes

10.7717/peerj.13806/supp-7 Supplemental Information 7 The ARRIVE guidelines 2.0 author checklist

## Additional Information and Declarations

### Data Availability

The following information was supplied regarding data availability: All the raw data are available at ProteomeXchange via iProX partner repository (https://www.iprox.cn/): PXD030691, IPX0003916000.
